# Assessment of Selected Intestinal Permeability Markers in Children with Food Allergy Depending on the Type and Severity of Clinical Symptoms

**DOI:** 10.3390/nu14204385

**Published:** 2022-10-19

**Authors:** Monika Niewiem, Urszula Grzybowska-Chlebowczyk

**Affiliations:** Department of Pediatrics, Faculty of Medical Sciences, Medical University of Silesia in Katowice, 40-752 Katowice, Poland

**Keywords:** food allergy, children, zonulin, atopic dermatitis

## Abstract

Background: Food allergy (FA) has a broad range of symptoms, and clinical manifestations may concern several reactions from one system or organ. Aim: The aim of the study was to assess intestinal permeability (IP) based on the analysis of serum zonulin and bacterial lipopolysaccharides (LPS) levels in children with FA, taking into account the pathomechanism of immune reaction, clinical symptoms of FA and their severity. Material and methods: The study comprised 103 patients aged 7–60 months (median 34); 49 children with IgE-mediated allergy and 25 children with non-IgE-mediated allergy; the reference group comprised 29 children with functional gastrointestinal disorders. IP markers were determined using ELISA. Results: There was no correlation between the severity of clinical symptoms and the level of IP markers in children with FA. Zonulin and LPS levels were significantly higher in children with FA and gastrointestinal symptoms. Zonulin levels in the subgroup of children with non-IgE-mediated FA and gastrointestinal symptoms were significantly higher than in the subgroup of children with IgE-mediated FA and these symptoms. The level of LPS was significantly higher in the subgroup with IgE-mediated FA and atopic dermatitis. Conclusions: Zonulin and LPS levels were significantly higher in children with FA compared to children from the reference group. Zonulin levels were significantly higher in children with non-IgE-mediated FA than in children with IgE-mediated FA.

## 1. Introduction

Over the past 20–30 years, dynamically increasing morbidity associated with atopic diseases, i.e., asthma, allergic rhinitis, and food allergies (FA), has been observed worldwide. Research data available in the literature confirm that the percentage of patients with atopic diseases reaches 40% in the entire population and shows a growing tendency [1].

According to the available data, signs and symptoms of food allergy develop in nearly 5% of the adult population and in almost 8% of children [2,3,4,5]. Eight key allergens are thought to trigger the process of food hypersensitivity, including products of animal origin, i.e., cow’s milk protein, hen’s egg protein, fish and shellfish, and products of plant origin, i.e., nuts, soybeans, and wheat. The World Health Organization even consider them as the “Big-8” food allergens that most commonly cause FA [3,4,5]. Importantly, many literature reports confirm the fact that the primary allergens causing clinical manifestations of FA in the pediatric population are cow’s milk proteins (2 to 3.2%) and chicken egg-white protein (2 to 2.5%) [3].

Clinical manifestations associated with FA may concern one system or organ, but more often, many systems are affected. Clinical symptoms are usually non-specific and diverse [1,2].

The development of symptoms in allergic reaction might be determined by one type of mechanism or, more frequently, a combination of overlapping mechanisms. Allergy symptoms additionally depend on the patient’s age, sex, ethnicity, target organs for clinically manifested reaction to food allergens, and simultaneous exposure to other food allergens (inhaled or contact) [6,7,8,9,10,11].

The immune pathogenesis of allergic diseases is strictly related to disorders of homeostasis associated with Th2 cells, which produce proallergic cytokines, such as interleukin 4 (IL-4), IL-5, and IL-13, and Th1 cells, which produce proinflammatory cytokines: IL-12, IL-18, interferon gamma, and tumour necrosis factor [12]. In atopic patients, the immune system responds to the allergen presence by producing higher amounts of antigen-specific IgE (IgE-mediated allergy). In patients with IgE-mediated allergy, the Th1/Th2 imbalance in favour of Th2 is observed. Other patients do not experience IgE-mediated reactions, but in their case, an excessive Th1 proinflammatory response is activated (non-IgE-mediated allergy) [13].

Because of increased intestinal permeability (IP) in the course of FA, allergens can cross the intestinal barrier and stimulate immune response in the submucosal layer. In subjects with a damaged intestinal barrier, i.e., functional gastrointestinal disorders affecting intercellular tight junctions, control over molecules that migrate to the bloodstream is partially or completely lost [14]. Studies have demonstrated that increased IP is associated with increased levels of some substances, including zonulin and bacterial lipopolysaccharide (LPS).

Zonulin is a protein that modulates the permeability of tight junctions between enterocytes [15,16]. The activation of the zonulin pathway results in a number of reactions leading to a significant rearrangement of the tight junctions. Ultimately, this reduces the density of the intestinal mucosa and results in an excessive trafficking of macromolecules through the intestinal mucosa, which may contribute to immunization and cause intestinal or non-intestinal inflammatory, autoimmune, and allergic diseases [15,16,17,18,19,20,21,22,23,24].

LPS are large molecules building the cell wall of Gram-negative bacteria, which are part of the intestinal barrier, namely the gut microbiome. Bacterial endotoxins are responsible for the development of chronic inflammation and the increase in the secretion of pro-inflammatory cytokines, leading to intestinal barrier damage and, consequently, increasing its permeability. Disorders of the gut microbiome can also increase zonulin release and modulate tight junctions between endothelial cells, increasing the severity of endotoxemia [25,26].

In pathological conditions, the level of LPS in the intestinal tissue and in the bloodstream is significantly elevated; the level of LPS is usually highest in the intestinal lumen colonized by intestinal bacteria and low or undetectable in the circulating plasma because the normally functioning intestinal epithelial layer forms an effective barrier against LPS migration [25,26]. However, if the intestinal barrier is defective, LPS and other water-soluble antigens can migrate from the intestinal lumen, causing an increase in LPS level in intestinal tissue and plasma [27].

## 2. Study Aim

The aim of the study was to assess IP based on the analysis of serum zonulin and LPS levels in infants and young children with FA, taking into account the pathomechanism of immune reaction, clinical symptoms of FA, and their severity.

## 3. Material and Methods

### 3.1. Patients

The study comprised 103 patients aged 7 months to 5 years (mean 32.7 ± 17.0; median 34 months); 59% of them were boys. The study group comprised 74 children with newly diagnosed FA, including 49 children with IgE-mediated allergy and 25 with non-IgE-mediated allergy, and the reference group comprised 29 children with functional gastrointestinal disorders. The examined children were diagnosed at the Gastroenterology Department of the Pediatrics Clinic, Medical University of Silesia, the Upper Silesian Child Health Centre in Katowice in Poland, between 2017 and 2020.

### 3.2. Research Methods

FA was diagnosed based on:

- Information gathered during interview with patients and their family;
- Results of laboratory tests, including measurements of: total IgE (tIgE) and specific IgE (sIgE) for selected food allergens;
- Diagnostic elimination test and food challenge.

**Figure 1:**
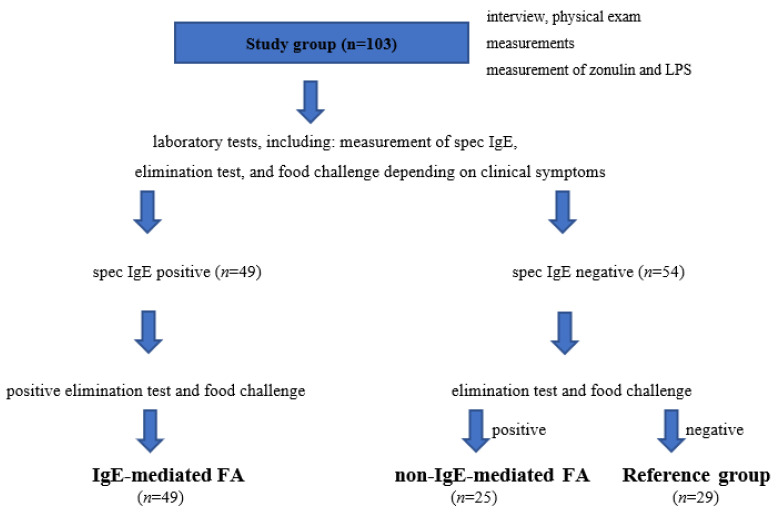
Study protocol and allocation of patients to the study and reference groups. FA, food allergy; LPS, bacterial lipopolysaccharides.

The study protocol is presented in Figure 1.

In patients from the study group (with newly diagnosed FA, not being on an elimination diet), we analysed the most common clinical manifestations of FA, the severity of clinical symptoms (considering the number of organs/systems involved), and the severity of atopic dermatitis (AD) based on the SCORAD index [28].

The SCORAD index is used for assessment of AD severity based on objective signs such as the extent of skin lesions (A) and their intensity (B) and subjective symptoms (C) including pruritus and sleep disorders reported by the patient/the child’s caregiver that occur during three previous days. The SCORAD index classifies AD into three severity categories: mild (<25 points), moderate (25 to 50 points), and severe (>50 points). The maximum possible score is 103 points (severe exacerbation of the disease), and the minimum score is 0 (currently no lesions). The severity of lesions is determined using the following formula: *SCORAD: A/5 + 7B/2 + C* [28]. The levels of zonulin and LPS were measured in all children from the study and reference groups. To measure zonulin and LPS levels in the children from the study and reference groups, an additional 2 mL of peripheral blood were collected to tubes without anticoagulants (to obtain clotted blood samples) during blood collection for tests associated with current hospitalization. The blood samples were centrifuged (10 min at 3000 RPM). Next, the serum was removed, separated into two Eppendorf tubes, frozen, and stored until testing. IP markers were determined using ELISA (enzyme-linked immunosorbent assay) at the Department of Pathophysiology, Faculty of Medical Sciences in Katowice. The quantitative assessment of serum zonulin levels was performed using a commercially available Zonulin ELISA kit (Immunodiagnostik AG, Bensheim, Germany), and LPS levels were quantitatively determined using an Enzyme-linked Immunosorbent Assay Kit for Lipopolysaccharide (Cloud-Clone Corp., USA,23603 W. Fernhurst Dr., Unit 2201, Katy, TX 77494).

The design of the medical experimental study was approved by the Bioethical Committee of the Medical University of Silesia in Katowice, decision no. KNW/0022/KB1/62/17 of 27 July 2017. The study was funded by the Medical University of Silesia in Katowice from the budget for the implementation of research, decision no. KNW-2-K34/D/8/N.

### 3.3. Statistical Analysis

The values of continuous variables were presented as means with standard deviation or medians with a quartile range (the lower and upper quartiles). The distribution of continuous variables was analysed with the Shapiro–Wilk test. Differences between the two groups were assessed using the Mann–Whitney U test for non-normally distributed variables or the Student’s *t*-test for normally distributed variables. Statistics for more than two groups were compared using the parametric analysis of variance or its non-parametric equivalent, the Kruskal–Wallis test. The homogeneity of variance was verified using Levene’s test.

The relationship between selected variables was evaluated with Spearman’s correlation test. Risk factors for increased zonulin and LPS levels (zonulin or LPS levels higher than two tertiles for all analysed patients) were assessed with univariate logistic regression, and its results were presented with odds ratio and confidence interval. The gathered data were processed using STATISTICA 13.0 (STATSOFT; Statistica, Tulsa, OK, USA) and SAS 9.3 (SAS Institute Inc., Cary, NC, USA).

## 4. Results

### 4.1. Most Common Clinical Manifestations

Children participating in the study were assessed in terms of clinical symptoms. Medical interviews focused on gastrointestinal and respiratory symptoms, skin conditions, and other symptoms. It should be pointed out that the reference group in the study comprised patients with functional gastrointestinal disorders, mainly functional diarrhoea and/or constipation, and therefore, these symptoms were reported on admission by nearly 90% of the subjects from this group. Gastrointestinal symptoms were found in 80% of children with non-IgE-mediated FA and 67% of children with IgE-mediated FA (Table 1).

Respiratory symptoms were present in fewer patients, i.e., six children with FA (three with IgE-mediated FA and three with non-IgE-mediated FA) and only one child from the reference group (Table 1). Skin conditions are frequently observed in pediatric patients with FA. Skin conditions were significantly more common in children with non-IgE-mediated FA than in those with IgE-mediated FA (Table 1). The observed skin signs and symptoms included: AD severity, pruritus, and urticaria not associated with infection or medication use.

We also assessed other symptoms, including weight loss, growth disorders, irritability/anxiety, and anorexia. No severe clinical manifestations of allergy such as anaphylaxis or food-protein-induced enterocolitis syndrome were diagnosed in any of the groups. The incidence of symptoms from other organs in children with IgE-mediated FA and non-IgE-mediated FA was similar (57% and 60%, respectively). In the reference group, five patients had eating disorders, one child had growth retardation, and another four patients showed irritability and anorexia. The incidence of other symptoms was significantly higher in children with FA than in the reference group (*p* < 0.05) (Table 1).

### 4.2. Severity of Clinical Symptoms Depending on the Number of Systems Affected

In both types of allergy, moderate clinical symptoms were most common (65% in IgE-mediated FA and 72% in non-IgE-mediated FA). Mild symptoms were more common in patients with IgE-mediated FA than those with non-IgE-mediated FA (35% vs. 28%). Severe clinical manifestations of allergy such as anaphylaxis or food protein-induced enterocolitis syndrome were not diagnosed in any of the groups. No significant differences were found in the distribution of data on the severity of FA between the studied subgroups of children. Analysis did not demonstrate any relationship between the severity of clinical symptoms and the levels of IP markers in the subgroups of allergic children.

The severity of clinical symptoms was also assessed based on the number of systems/organs affected. In both types of allergy, reactions involving many organs (two to four systems) were most common, and of these, the involvement of 2–3 systems concerned nearly 61% of patients with IgE-mediated FA and 80% of patients with non-IgE-mediated FA. Allergic reaction involving one system/organ was more common in patients with IgE-mediated FA than in those with non-IgE-mediated FA (33% vs. 12%), while the involvement of four systems was the least common and concerned 8% of children with non-IgE-mediated FA and 6% of children with IgE-mediated FA. Significant differences were found in the type and number of systems/organs affected within individual subgroups of children (IgE-mediated FA *p* = 0.001; non-IgE-mediated FA *p* < 0.05). However, the comparison of the studied groups (IgE-mediated FA vs. non-IgE-mediated FA) did not reveal any relationship between the number of systems/organs affected. Analysis did not demonstrate any relationship between the number of affected systems/organs and the level of IP markers in the subgroups of allergic children.

### 4.3. Severity of AD Assessed with SCORAD

The severity of AD was assessed by SCORing Atopic Dermatitis (SCORAD) [28]. AD in children with IgE-mediated FA was mild (68%) or moderate (29%), and only one patient had a severe disease. The severity distribution of AD was similar in children with non-IgE-mediated FA and those with IgE-mediated FA. Differences in the severity of AD were not statistically significant. Another analysis did not show any significant trend in the proportions of the severity of AD expressed by the SCORAD index (*p* = 0.9).

Analysis of zonulin levels in the subgroups of children with IgE-mediated FA depending on the severity of AD showed no significant differences, and similar findings were made for children with non-IgE-mediated FA. Comparison of LPS levels in the subgroups of children with IgE-mediated FA and different severity AD showed no significant differences, and similar findings were made for children with non-IgE-mediated FA.

### 4.4. Levels of Selected IP Markers—Comparison of All Children with FA and Reference Group and Depending on the Immune Pathomechanism of FA

We analysed differences in zonulin and LPS levels between all children with FA and the reference group. Zonulin and LPS levels were significantly higher in children with FA compared to children from the reference group. LPS levels were also significantly higher in children with FA compared to those from the reference group (Table 2). The mean zonulin levels were significantly higher in children with non-IgE-mediated FA than in those with IgE-mediated FA (*p* < 0.05). There were no significant differences in LPS levels between children with IgE-mediated FA and non-IgE-mediated FA (Table 2).

#### 4.4.1. Relationship between the Levels of Selected IP Markers and Clinical Manifestations in Children with IgE-Mediated and Non-IgE-Mediated FA

Zonulin levels were significantly higher in children with FA and gastrointestinal symptoms (*p* < 0.01) than in the reference group of children with gastrointestinal symptoms. LPS levels were also significantly higher in children with FA and gastrointestinal symptoms (*p* = 0.001) than in the reference group.

There were no significant differences in zonulin levels between children with IgE-mediated FA and non-IgE-mediated FA depending on the analysed clinical symptoms. LPS levels were significantly higher in the subgroup of children with IgE-mediated FA and AD (*p* < 0.01) (Table 3).

#### 4.4.2. Relationship between the Levels of Selected IP Markers and Gastrointestinal Symptoms—Comparison of Children with IgE-Mediated and Non-IgE-Mediated FA

Mean zonulin level in the subgroup of children with non-IgE-mediated FA and gastrointestinal symptoms was significantly higher (*p* < 0.05) than in the subgroup of children with IgE-mediated FA. There were no significant differences in LPS level between these two subgroups (Table 4).

### 4.5. Dependent Variables Influencing the Risk of Increased Zonulin and LPS Levels in the Analysed Groups of Children

Both types of FA were associated with a risk of increased levels of IP markers. The risk of increased zonulin and LPS levels was many times higher in children with non-IgE-mediated FA (Table 5).

## 5. Discussion

FA has a broad range of symptoms. The most common clinical manifestations of FA are gastrointestinal and/or skin conditions [29,30,31,32]. Moreover, the clinical picture of FA changes with age (allergic march), which results from the anatomical and functional maturation of organs exposed to a harmful allergen. In our study, the most frequently reported symptoms in children with IgE-mediated FA and non-IgE-mediated FA were those related to the skin (71% and 92%, respectively). Studies carried out in the 1950s revealed that the most common symptom was AD (43% of patients), followed by gastrointestinal and respiratory symptoms and general manifestations [33,34]. Mayer et al. also indicated the predominance of AD among the symptoms of FA [29]. Rowicka et al. reported different observations. In their study, 42% of children with cow’s milk protein allergy had gastrointestinal symptoms, and only 17% had skin conditions [30]. A Brazilian observational cross-sectional study also demonstrated the dominance of gastrointestinal symptoms among examined children up to 2 years of age, and they concerned as many as 89% of patients [31]. Our study concerned older children, and skin conditions were the most common clinical manifestations.

Different studies have indicated the sequential progression of atopic diseases, which is defined as the allergic march. Usually, an allergy begins with a hypersensitivity to food and progresses to AD and, over time, to hay fever and even to bronchial asthma. The attenuation of FA may be accompanied by a stronger reaction to inhaled allergens. The risk of developing allergic march has been linked with genetic predispositions and extrinsic factors (i.e., stress, diet, infections, exposure to tobacco smoke, and smog) [35,36,37].

In our study, both measured markers of IP, zonulin and LPS, were significantly higher in children with FA than in the reference group. It should be emphasized that the allergic disease, regardless of the pathomechanism of the immune reaction, determined the increase in IP, which is strictly related to the abnormal function of the intestinal barrier and the intestinal microbiome involved in its formation. Recent reports have revealed lower diversity of gut microbiota in children diagnosed with allergies. Studies have demonstrated that allergic patients were less often colonized with *Bacteroidetes*, *Bifidobacterium*, and *Lactobacillus* [38,39]. Importantly, the differences in the composition of microbiota between healthy children and those with atopic disease are already present in early infancy before the clinical manifestation of allergy, which was confirmed in a study by Kalliomaki et al. [40]. Since an increased count of Gram-negative bacteria, a component of the gut microbiome, is the source of LPS, an association between the secretion of LPS and gut microbiome disorders was found. Bacterial endotoxins are responsible for the development of chronic inflammation and the increase in the secretion of pro-inflammatory cytokines. This leads to intestinal barrier damage and, consequently, increases its permeability [41,42,43]. Gut microbiota disorders can also increase zonulin release and modulate tight junctions between cells, increasing the severity of endotoxemia [16].

Our study analysing IP depending on the pathomechanism of immune response in FA revealed significantly higher levels of zonulin in children with newly diagnosed non-IgE-mediated FA than in children with IgE-mediated FA and insignificantly higher levels of LPS in children with non-IgE-mediated FA. Both types of FA are associated with a risk of increased levels of IP markers. The risk of increased zonulin and LPS levels is many times higher in children with non-IgE-mediated FA. Increased IP in patients with non-IgE-mediated FA may be caused by chronic inflammation, which has a greater extent and more serious consequences than in the case of anomalies associated with IgE-mediated FA. Non-IgE-mediated FA has a less pronounced onset, gastrointestinal symptoms are chronic, and the advanced disease leads to protein-losing enteropathy resulting in malabsorption and iron-deficiency anaemia [44].

Because gastrointestinal symptoms in our study were found in the majority of patients from the reference group (90%) and over 70% of children with FA, a comparative analysis of zonulin and LPS levels was performed, taking into account the presence of gastrointestinal symptoms in both of these groups. Analysis demonstrated that the levels of IP markers were significantly higher in children with FA and gastrointestinal symptoms than in children from the reference group. Considering the pathomechanism of immune response in FA, levels of zonulin measured in the subgroup of children with non-IgE-mediated FA and gastrointestinal symptoms were significantly higher (*p* = 0.03) than in the subgroup of children with IgE-mediated FA and these symptoms. Among children with AD, the level of LPS was significantly higher in the subgroup of children with IgE-mediated FA. This might be associated with a strong dominance of skin conditions in the group of examined children. Significantly increased serum zonulin levels in children with AD were reported by Sheen et al. [45].

A study by Kalach et al. also found that IP was increased in 80% of children with gastrointestinal symptoms and was significantly higher in children with cow’s milk protein allergy and digestive symptoms than those with other clinical manifestations [46]. Järvinen et al. reported increased IP in asymptomatic children on long-term elimination diets [47]. Whether the increased IP observed in patients with FA is the primary disorder predisposing them to FA or a consequence of ongoing symptomatic or asymptomatic exposure to allergic triggers remains an open question. One hypothesis assumes that increased IP may be a genetic trait in patients with FA as a result of a chronic allergic process and damage to the intestinal mucosa or an unintentional ingestion of small amounts of food allergen [48,49].

Our analysis demonstrated no relationship between the severity of clinical symptoms and the concentration of IP markers in the subgroups of allergic children. Moreover, no relationship was found between the number of systems affected and the levels of IP markers in children diagnosed with FA. An increase in IP associated with greater severity of clinical symptoms of FA (evaluated by lactulose/mannitol ratio) was only reported by Ventura et al. [50].

The present study also found no correlation between the severity of AD assessed with SCORAD and the levels of zonulin or LPS. Increased severity of symptoms measured with SCORAD was associated with significantly higher total IgE values in all children with AD and in children with IgE-mediated FA. However, the previously cited study by Sheen et al. found no relationship between the severity of AD and total IgE or eosinophils [45]. Instead, their analysis covering older children revealed significantly increased serum zonulin levels depending on the presence and severity of AD. In our study, the concentration of zonulin was normal, but we found increased levels of LPS in children with IgE-mediated FA and AD independent of the severity of clinical symptoms [45]. Perhaps age was a factor influencing the concentration of IP markers. Some studies have confirmed that the immature intestinal barrier is involved in the pathomechanism of atopic diseases, and this problem is currently under investigation. The formation of the human intestinal barrier is gradual. It starts in the foetal period and continues after birth and throughout the neonatal and infancy period [51,52,53]. Corpeleijn et al. also emphasized that increased IP is physiological in neonates and may play an important role in the absorption of larger molecules of nutrients from breast milk and induce systemic tolerance in the body [54]. In the treatment of IP conditions, an appropriate diet and probiotic supplementation are recommended. The diet is aimed at restricting processed and high-fat food [55]. In addition, glutamine supplements are recommended [55,56]. It has been demonstrated that vitamin A and its derivatives control the growth and differentiation of intestinal cells, while vitamin A deficiency is associated with increased susceptibility to infections [57]. There are papers describing the importance of vitamin D in prevention and treatment of IP conditions [58]. Reports available in the literature mention the role of vitamin D deficiency and decreased levels IP markers in the intensive care setting, which suggest early manifestations of IP [59]. Moreover, vitamin D deficiency promotes intestinal barrier dysfunction in inflammatory bowel diseases [60].

## 6. Conclusions

Zonulin and LPS levels were significantly higher in children with FA compared to children from the reference group. Zonulin levels were significantly higher in children with non-IgE-mediated FA (*p* < 0.05) than in children with IgE-mediated FA. Increased levels of IP markers were found more frequently in patients with FA and the presence of skin conditions and gastrointestinal symptoms.

This is the first study that analysed the levels of two markers of IP, zonulin and LPS, depending on the type and severity of clinical symptoms in pediatric patients with FA determined by different pathomechanisms of immune response.

FA remains an important and difficult problem in everyday medical practice. This is due to the variety of immune pathomechanisms of FA as well as the absence of specific allergy symptoms in response to the most common food allergens. Symptoms of allergy are non-specific; their progression dynamics is varied and may depend on the type of food (cooking and other methods of processing may change the sensitizing properties of some allergens, e.g., milk and its products) and the amount of allergen ingested. Another difficulty results from the potential exposure to other factors that can modulate symptoms. Poor availability of reliable laboratory tests that would confirm the diagnosis of allergy is also a serious problem.

## Figures and Tables

**Table 1:** Incidence of analysed systemic symptoms in different groups.

Clinical Symptoms	Group				*p*	
	1	2	3	4	1 vs. 4	2 vs. 3
	Allergy Total	IgE-Mediated FA	Non-IgE-Mediated FA	Reference Group		
	n = 74	n = 49	n = 25	n = 29		
Gastrointestinal	53 (71%)	33 (67%)	20 (80%)	26 (90%)	0.05	0.1
n (%)						
Respiratory	6 (8%)	3 (6%)	3 (12%)	1 (3%)	0.3	0.4
n (%)						
Skin conditions	58 (78%)	35 (71%)	23 (92%)	0	n/a	<0.001
n (%)						
Other	43 (54%)	28 (57%)	15 (60%)	10 (34%)	<0.05	0.1
n (%)						

FA, food allergy; n/a, not applicable.

**Table 2:** Zonulin and LPS levels—comparison of all children with FA and reference group depending on the immune pathomechanism of FA.

Marker		Allergy Total	Reference Group	*p*	IgE-Mediated FA	Non-IgE-Mediated FA	*p*
		n = 74	n = 29		n = 49	n = 25	
Zonulin (ng/mL)	Mean ± SD	35 ± 13	23 ± 13	<0.0001	33 ± 12	39 ± 14	<0.05
	Median	34	19		32	38	
	IQR	27–43	13 ± 34		27–40	29–47	
LPS (ng/mL)	Mean ± SD	904 ± 258	675 ± 385	<0.0001	896 ± 253	919 ± 271	0.5
	Median	919	601		907	1005	
	IQR	755–1111	402–845		755–1071	773–1147	

FA, food allergy; LPS, bacterial lipopolysaccharides; IQR, interquartile range.

**Table 3:** Levels of selected IP markers in children with IgE-mediated and non-IgE-mediated FA and different clinical manifestations.

**Marker**		**IgE-Mediated** **FA**		***p***	**Non-IgE-Mediated** **FA**		***p***
		**Gastrointestinal Symptoms**			**Gastrointestinal Symptoms**		
		**Yes (n = 33)**	**No (n = 16)**		**Yes (n = 20)**	**No (n = 5)**	
Zonulin (ng/mL)	Mean ± SD	32 ± 12	35 ± 12	0.4	39 ± 12	41 ± 20	0.9
	Median	32	35		37	42	
	IQR	25–41	28–40		31–46	24–47	
LPS (ng/Ml)	Mean ± SD	864 ± 290	961 ±140	0.2	931 ± 291	873 ± 188	0.3
	Median	872	935		1070	812	
	IQR	720–1071	869–1073		716–1170	804–1005	
**Marker**		**IgE-Mediated** **FA**		***p***	**Non-IgE-Mediated** **FA**		***p***
		**Respiratory Symptoms**			**Respiratory Symptoms**		
		**Yes (n = 3)**	**No (n = 46)**		**Yes (n = 3)**	**No (n = 22)**	
Zonulin (ng/Ml)	Mean ± SD	26 ± 12	33 ± 12	0.1	31 ± 4	40 ± 14	0.1
	Median	25	33		29	42	
	IQR	21–32	27–41		29–36	33–47	
LPS (ng/mL)	Mean ± SD	529 ± 379	920 ± 229	0.1	1047 ± 128	902 ± 282	0.5
	Median	665	926		1053	951	
	IQR	101–823	781–1076		916–1173	658–1147	
**Marker**		**IgE-Mediated** **FA**		***p***	**Non-IgE-Mediated** **FA**		***p***
		**Skin Conditions**			**Skin Conditions**		
		**Yes (n = 35)**	**No (n = 14)**		**Yes (n = 23)**	**No (n = 2)**	
Zonulin (ng/mL)	Mean ± SD	33 ± 12	32 ± 12	0.8	40 ± 14	33 ± 5	0.5
	Median	32	30		41	33	
	IQR	26–39	27–43		2–47.0	29–37	
LPS (ng/mL)	Mean ± SD	1031 ± 224	824 ± 241	<0.01	1057 ± 199	908 ± 658	0.3
	Median	1094	854		1057	1005	
	IQR	898–1160	731–969		916–1198	261–1252	
**Marker**		**IgE-Mediated** **FA**		***p***	**Non-IgE-Mediated** **FA**		***p***
		**Other Symptoms**			**Other Symptoms**		
		**Yes (n = 28)**	**No (n = 21)**		**Yes (n = 15)**	**No (n = 10)**	
Zonulin (ng/mL)	Mean ± SD	30 ± 11	36 ± 12	0.1	38 ± 13	42 ± 14	0.3
	Median	31	36		36	43	
	IQR	23–39	27–43		29–45	33–48	
LPS (ng/mL)	Mean ± SD	865 ± 283	937 ± 207	0.1	921 ± 309	917 ± 218	0.5
	Median	854	964		1053	951	
	IQR	690–1100	872–1071		773–1173	658–1112	

FA, food allergy; LPS, bacterial lipopolysaccharides; IQR, interquartile range.

**Table 4:** Levels of selected IP markers vs. presence of gastrointestinal symptoms—comparison of children with IgE-mediated and non-IgE-mediated FA.

Marker		Gastrointestinal Symptoms					
		Yes		*p*	No		*p*
		IgE-Mediated FA	Non-IgE- Mediated FA		IgE-Mediated FA	Non-IgE- Mediated FA	
		n = 33	n = 20		n = 16	n = 5	
Zonulin (ng/mL)	Mean ± SD	32 ± 12	38.8 ± 12.2	<0.01	35 ± 12	41 ± 20	0.4
	Median	32	37.4		35	42	
	IQR	25–41	31.0 ± 45.7		28–40	24–47	
LPS (ng/mL)	Mean ± SD	864 ± 290	931.1 ± 290.0	0.1	961 ± 140	873 ± 188	0.7
	Median	872	1070.0		935	812	
	IQR	720–1071	715.7–1169.5		869–1073	804–1005	

FA, food allergy; LPS, bacterial lipopolysaccharides; IQR, interquartile range.

**Table 5:** Risk factors for increased zonulin level (>2 tertiles 41 ng/mL) and increased LPS level (>2 tertiles 1071 ng/mL) in the analysed groups of children with IgE-mediated and non-IgE-mediated food allergy.

Dependent Variable	Group	OR (95% CI)
Increased zonulin level (ng/mL)	Reference group	1
	non-IgE-mediated FA	12.5 (2.4–64)
	IgE-mediated FA	4.4 (0.9–21.2)
Increased LPS level (ng/mL)	Reference group	1
	non-IgE-mediated FA	10.6 (2.1–54.6)
	IgE-mediated FA	4.9 (1–23.4)

FA, food allergy; LPS, bacterial lipopolysaccharides.

## Data Availability

The statistical analysis and database used to support the findings of this study may be released upon application to the Medical University of Silesia, Department of Pediatrics, who can be contacted by the corresponding author.
